# 2069. Exploring the Barriers and Enablers of Safe Oral Sexual Practices among Transgender Women of Malaysia: A Qualitative Study on STI Transmission

**DOI:** 10.1093/ofid/ofad500.139

**Published:** 2023-11-27

**Authors:** Lahari Ajay Telang, Hezreen Shaik Daud, Aoife Cotter, Abdul Rashid

**Affiliations:** Penang International Dental College, Bukit Mertajam, Pulau Pinang, Malaysia; Penang International Dental College, Bukit Mertajam, Pulau Pinang, Malaysia; 1Centre for Experimental Pathogen Host Research (CEPHR), University College Dublin, Belfield, Dublin 4, Ireland 5Department of Infectious Diseases, Mater Misericordiae University Hospital, Eccles St, Dublin 7, Ireland, Dublin, Dublin, Ireland; RCSI-UCD Malaysia Campus, Georgetown, Pulau Pinang, Malaysia

## Abstract

**Background:**

Transgender women (TGW) in Malaysia experience unique social and interpersonal challenges that contribute to their risk of acquiring HIV and sexually transmitted infections (STIs). (Fig 1)The study aimed to understand the experiences of this vulnerable population, barriers and enablers of safe sexual practices relating to oral transmission of STIs.

**Fig 1:**
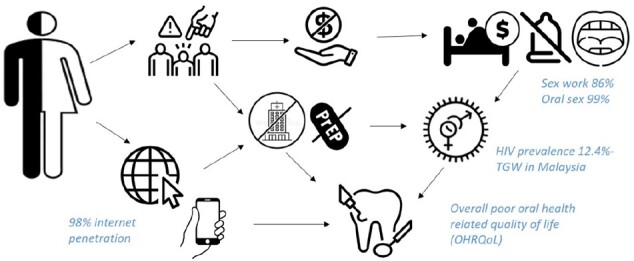
Challenges faced by Transgender women in Malaysia

**Methods:**

Participants were recruited through a snow-balling method of sampling with the help of TGW community advocates. Theories of Health Belief Model and Andersen’s Behaviour Model of health services use were used to devise the interview guide. Semi-structured in-depth interviews and Focus group discussion with participants gave insights into the needs of the community. (Fig 2) The data obtained was transcribed, coded and subjected to thematic analysis using the constructs of the Information, Motivation and Behavioural skills (IMB) theory. Results are presented as n (%) or median (interquartile range, unless otherwise stated).

**Fig 2:**
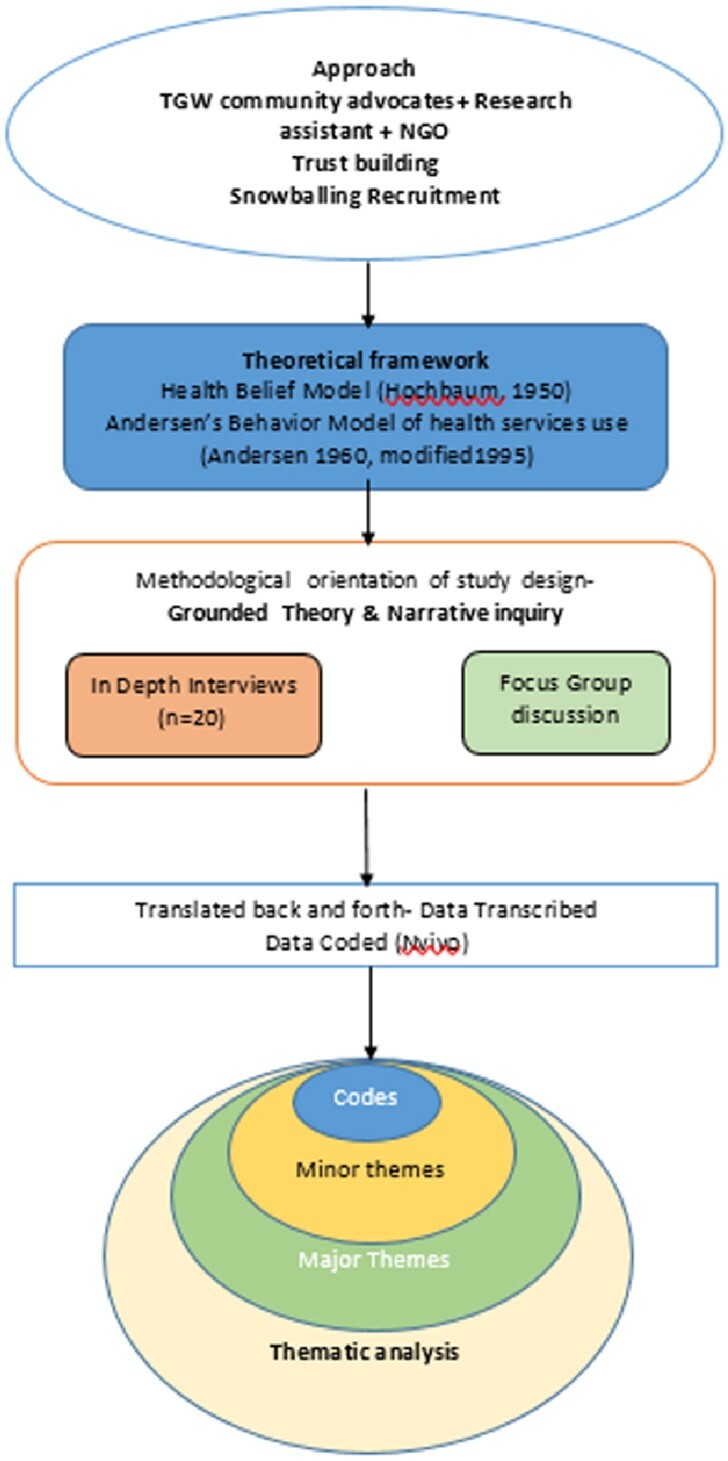
Methodology Flowchart -Qualitative analysis

**Results:**

Participant characteristics are outlined in table 1. The major themes that emerged through qualitative analysis are highlighted (Fig 3). The results identified gaps in awareness of oral transmission of STIs among TGW and lack of condom use during oral sex. Self-medication / non-professional treatments and lack of utilization of dental services was a concern.

**Table 1:**
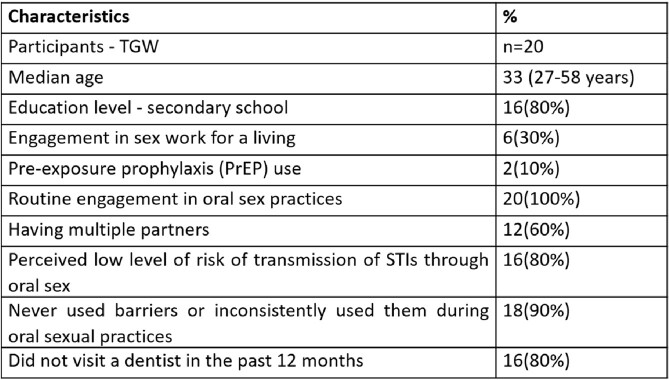
Participant characteristics

**Fig 3:**
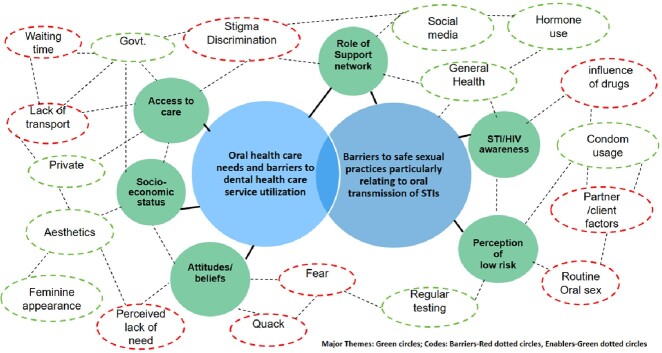
Thematic analysis map of In-depth Interviews with Major themes and Minor themes indicating Barriers and Enablers

**Conclusion:**

The importance of social support in positively influencing health promotion as well as improving health care and dental care utilization was highlighted. The areas of concern identified through this qualitative research will inform the design and development of an educational intervention aimed at addressing these concerns and providing support. Developing a customised, culturally sensitive, peer reviewed educational intervention that will be delivered through a widely popular social media platform is proposed.

**Disclosures:**

**All Authors**: No reported disclosures

